# Supplementary material for: Patterns of Intron Gain and Loss in Fungi
Source: PLoS Biol. 2004 Nov 30;2(12):e422. doi: 10.1371/journal.pbio.0020422 (PMC532390; doi:10.1371/journal.pbio.0020422)
Supplement: Table S1 — Also available at http://genes.mit.edu/NielsenEtAl/. (4.3 MB ZIP). [file pbio.0020422.st001.zip › NielsenEtAl/html/1209.html]

AN9157.1.NCU07926.1.MG09393.1.FG02555.1


```
 CLUSTAL W (1.82) Multiple Sequence Alignments - Introns Inserted


Sequence 1: AN9157.1	628 aa
Sequence 2: FG02555.1	631 aa
Sequence 3: NCU07926.1	651 aa
Sequence 4: MG09393.1	632 aa
Alignment Length: 659 aa
Number Identitical Residues: 349 aa
Alignment Score (without introns) 16829


MG09393.1 	---MADAITEGAAKLQ--------------LDEETGEMVSKGELKKRMQKRAKKAAQAK-
NCU07926.1	---MTDAVVDAAAKMQLADAPVDPNAPKLVLDEETGEMVSKGELKKRLAKRAKKAIKEKN
FG02555.1 	MSDPVAEVTEATVKLV--------------LDEVTGEQVTRNELKKRTQKRAKKAAAQAS
AN9157.1  	---MTEALAEATAKLV--------------LDEETGEMVSKNELKKRTQKRAKKAAAAAA
          	    .  :.:.:.*:               *** *** *::.*****  ******     

MG09393.1 	---AKEAAQATAGDAEKKAPAKPAAKPEEVVIDADAMFKQGFLDEVFRNRPMKPVFTRFP
NCU07926.1	KDDKKNVAATTAAAQDGATPKKQTPKPEEAPIDTQAMFKQGFLNDVYNERPVKPVFTRFP
FG02555.1 	-----REEKAKNQEANPKPAAPKPKAQEPTQLDPDAMFKQGFLADVYKERPVKPVVTRFP
AN9157.1  	-----KANQAQKP-ATPKPKT-EVKPAEETPLDPDAMFKQGFLANVFRERPMKPVLTRFP
          	     .   :        .        * . :*.:******** :*:.:**:***.****

MG09393.1 	PEPNGFLHIGHAKAIAINFGFARHHGGQC~YLR~YDDTNPEKEEEKYFTSIESMVRWLG~
NCU07926.1	PEPNGFLHIGHAKAIAINFGFAKYHGGNC~YLR~FDDTNPEAEEEIYFTAIEDTVRWLG~
FG02555.1 	PEPNGYLHLGHAKAIAIDFGFARYHGGKT0ILR2FDDTNPDEEEEIYFEWILKIIRWLG1
AN9157.1  	PEPNGYLHLGHCKAIAVNFGFARYHGGET0ILR~FDDTNPEAEEEKYFTAIEDIIQWLG1
          	*****:**:**.****::****::***:   ** :*****: *** **  * . ::*** 

MG09393.1 	FSPYKITYSSDNFDKLYELAEKLINLGKAYVCKCDDAEIKLQRGGEK-GKSPRYRCAHAD
NCU07926.1	FTPYKITYSSDNFQKLYDLAEKLIGLDKAYVCHCNDDEIKLQRGGEK-GSSPRFRCKHAE
FG02555.1 	FEPSAITHSSDNFQKLFDLAKELIKKDKAYVCHCNEAEIKLQRGGKEGKEGPRYRCAHAE
AN9157.1  	FKPARITHSSDHFQKLYEHAEKLIQLGKAYVCHCSDTEIKLQRGGE--NHGARYRCKDAE
          	* *  **:***:*:**:: *::**  .*****:*.: ********:    ..*:** .*:

MG09393.1 	QTPEDNLAQFRDMRDGKYKEREAFLRMKQDITDGNPQMW~DLAAYRVKK--AEHHRTGDK
NCU07926.1	QSVEENLQKFRDMRDGKYKPREAFLRMKQDITDGNPQMW~DLAAYRVLD--KPHHRTGSQ
FG02555.1 	QDVETNLQKFQDMHDGKYAPQTAFLRMKQDITNGNPQMW~DLAAYRIPKNQKVHHRAP-E
AN9157.1  	QDVETNLKKFRDMKDGKYEPQTAFLRMRQDIDNPNPQMW0DLAAYRVLK--KEHHKAP-G
          	*  * ** :*:**:****  : *****:*** : ***** ******: .    **::   

MG09393.1 	WKIYP0TYDFT~HCLCDSFEGITHSLCTTEFIQSRQSYEWLNTTLGVYEPQQREYG~RLG
NCU07926.1	WKIYP~TYDFT~HCLCDSFEGITHSLCTTEFVLSRVSYEWLNKSLEVYEPMQREYG~RLS
FG02555.1 	WKIFP~TYDFT~HCLCDSFEGITHSLCTTEFILSRESYEWLNKSLEVYEPMQREFG1RLN
AN9157.1  	WVIFP~TYDFA0HCLCDSFEGITHSLCTTEFVLSRESYEWLNSTLGVYEPMQREYG~RLN
          	* *:* ****: *******************: ** ******.:* **** ***:* **.

MG09393.1 	LGGTVLSKRKILKLVELGHVREWDDPRLYTLTAIKRRGVPPGAILEFVNELGVTTAATVI
NCU07926.1	LSGTVLSKRKLKELVDRKIVRGWDDPRLYTLIAVRRRGVPPGAILEFVNELGVTTSNSII
FG02555.1 	VSGTIMSKRALKKLVEGGYVRAWDDPRLYTLIAIKRRGIPPGALLAFINELGVTTAKTII
AN9157.1  	VSGTIMSKRGLKKLVDGGYVRAWDDPRLYTVIALRRRGVPPEAILSFVNELGVTTANSVI
          	:.**::*** : :**:   ** ********: *::***:** *:* *:*******: ::*

MG09393.1 	QIKRFEQTIRRYLERTVPRLMMVLDPLRVVIEDAD--DLDGKPLSIPFSSKVPEMGSHDL
NCU07926.1	QIVRFEQTIRRYLERTVPRLMLVLDPVPVVIEDAD--EFDGSELTVPFSSKNAAMGDHKI
FG02555.1 	QIARFEQTVRRYLENTVPRLMLVLDPIPVTIEDHE----ETVELEVPYSPKDPKFGTHKV
AN9157.1  	NIARFEQSIRTYLESRVPRLMLVLDPLPVVIEDFDTLSAEQLNLDIPFSPKDPAMGSHQV
          	:* ****::* ***  *****:****: *.*** :: . :   * :*:*.* . :* *.:

MG09393.1 	KFTKTVYIDRSDFREVDSKDYFRLAPGKPVGLLNVPYPIKVKSFSKDKATGEVTEVRAEF
NCU07926.1	KFTKTVYIDRSDFREVDSKDYFRLAPGKTVGLLQAPFPIKATSFTKDETTGKVTEIRAVF
FG02555.1 	RFTKTVYIDRSDFREEDIKGYFRLAPGKTVGLLNAPHPIKATSFEKD-ESGKVTSIKAVF
AN9157.1  	GFTKTVYIDRSDFREEDAKGYFRLAPGKSVGLWKAPYPIKATTFTKD-ADGKITEVRAVL
          	 ************** * *.********.*** :.*.***..:* **   *::*.::* :

MG09393.1 	DREKKKPKAYIQWVP-EGSRKVEARVYNPLFKSESPDSVEGGFLADINPESQIIYPDALL
NCU07926.1	DRETKKPKTFIQWVGTDGSRKCEVRVYNQLFKSENPAAAEGGWLSDINPESEVVYPDALI
FG02555.1 	DKES-KPKTYIQWVP-EGSIKLEARVQSPLFKSDDPTAVEGGFLNDINPDSEVVYPEALV
AN9157.1  	DKDGGKPKTYIHWVP-EGSRKLEVRIHDQLFKSDEPAAAEGGFLADINPNSETIYADAMI
          	*::  ***::*:**  :** * *.*: . ****:.* :.***:* ****:*: :*.:*::

MG09393.1 	ESGFEEVRRRAPWPEAAGESELGKGGPESVRFQATRVAYF0AMDSDSTEDKIVLNRIVSL
NCU07926.1	ESGFDEVKRRAPWPEAAGESELGKGGPESVRFQAMRVAYF0ALDKESTDDKIVLNRIVSL
FG02555.1 	EEGFNEVRQRAPWPVTAGETNEAS-GPESVRFQAMRVAYF0AMDSDSTDDKIVLNRIVSL
AN9157.1  	ESGFDEVRRRAPWPEAAGETSEGTPRPETVRFQGMRVAYF~AMDSDSTDSHVVLNRIVSL
          	*.**:**::***** :***:. ..  **:****. ***** *:*.:**:.::********

MG09393.1 	KEDAGKN
NCU07926.1	KEDKEKN
FG02555.1 	KEDREKN
AN9157.1  	KQDTGKA
          	*:*  *
```
